# Some metabolic issues should not be neglected when using citrate for continuous renal replacement therapy!

**DOI:** 10.1186/s13054-015-0766-3

**Published:** 2015-02-06

**Authors:** Rita Jacobs, Patrick M Honore, Herbert D Spapen

**Affiliations:** ICU Department, Universitair Ziekenhuis Brussel – Vrije Universiteit Brussel, 101 Laarbeeklaan, 1090 Jette, Brussels, Belgium

**Keywords:** ᅟ

We read with great interest the paper by Schilder and colleagues on citrate anticoagulation versus systemic heparinisation (CASH) [1] and its related commentary [2], but would like to comment on two important metabolic issues.

First, in-circuit ionized calcium was not monitored in the CASH trial. Keeping postfilter ionized calcium within tight limits, however, results in more optimal anticoagulation, improves filter lifespan, and better regulates buffer supply. When studying this approach, we obtained a longer median filter survival time than that in the CASH study (98.4 versus 46 hours) [3]. We also infused calcium through a dedicated central venous line. As a result, the remaining citrate was not deactivated and its reinjection allowed continuous flushing of the dialysis catheter [3].

Second, in the presence of similar citrate and chloride flows, only a 2% incidence of metabolic alkalosis was observed in the regional citrate anticoagulation arm of the CASH trial whereas it occurred in all of our study patients after 48 hours [3]. In fact, an overly simplified definition of metabolic alkalosis (pH >7.5) was used in the CASH study [1]. Applying pH cutoff values of 7.5 and 7.45 identified metabolic alkalosis only in <1% and 12% of our study population, respectively [4]. We used the Stewart equation for evaluating the interacting impact of citrate, chloride, bicarbonate buffer and respiratory (over)compensation on acid–base status [5].

When calculating strong ion differences for patients receiving regional citrate anticoagulation in the CASH protocol, in particular at higher blood flow and thus higher citrate substitution, a substantially higher incidence of metabolic alkalosis would be noticed.

## Abbreviation

CASH: Citrate anticoagulation versus systemic heparinisation
